# Clinical presentation and diagnostic approach in cases of genitourinary tuberculosis

**DOI:** 10.4103/0970-1591.42626

**Published:** 2008

**Authors:** Rakesh Kapoor, M. S. Ansari, Anil Mandhani, Anil Gulia

**Affiliations:** Department of Urology and Renal Transplantation, Sanjay Gandhi Postgraduate Institute of Medical Sciences, Rai Bareli Road, Lucknow - 226 014, UP, India

**Keywords:** Diagnosis, genitourinary system, GUTB, tuberculosis

## Abstract

**Objective:**

We herein describe the various modes of presentation in genitourinary tuberculosis (GUTB) and a simple diagnostic approach to it.

**Materials and Methods:**

We made a literature search through Medline database and various other peer-reviewed online journals to study the various modes of presentation in GUTB. We reviewed over 100 articles published in the last 10 years (1998 -- 2007), which were tracked through the key words like GUTB and extrapulmonary tuberculosis.

**Results:**

GUTB has varied presentation and the most common way of presentation is in the form of irritative voiding symptoms, which are found in more than 50% of the patients. The usual frequency of organ involvement is: kidney, bladder, fallopian tube, and scrotum. The usual tests used to diagnose GUTB are the demonstration of mycobacterium in urine or body fluid and radiographic examination. Intravenous urography (IVU) has been considered to be one of the most useful tests for the anatomical as well as the functional details of kidneys and ureters. In cases of renal failure, MRI can be used. Newer examinations such as radiometric liquid culture systems (i.e., BACTEC^®^, Becton Dickinson, USA) and polymerase chain reaction (PCR) give rapid results and are highly sensitive in the identification of mycobacterium.

**Conclusion:**

GUTB can involve any part of the genitourinary system and presentation may vary from vague urinary symptoms to chronic kidney disease. Newer tests like radiometric liquid culture systems and polymerase chain reaction give rapid results and carry high diagnostic value.

## INTRODUCTION

Tuberculosis continues to be an important public health problem in our country. The World Health Organization (WHO) estimates that the largest number of new TB cases in 2005 occurred in the South-East Asia Region, which accounted for 34% of incident cases globally. It is estimated that 1.6 million deaths resulted from TB in 2005.[1] No reliable epidemiological data are available from India regarding its prevalence. Timely diagnosis and treatment will prevent the late sequelae of this disease, like nonfunctioning kidney and thimble urinary bladder. Here, we discuss the clinical presentation and diagnosis of genitourinary tuberculosis.

Genitourinary tuberculosis (GUTB) is the second most common form of extrapulmonary tuberculosis after lymph node involvement.[2] Kidney is usually the primary organ infected in urinary disease, and other parts of the urinary tract become involved by direct extension. Epididymis in men and fallopian tubes in women are the primary sites of genital infection.[3]

## CLINICAL PRESENTATION

The development of genitourinary tuberculosis usually arises from the spread of pulmonary tuberculosis, where mycobacterium spread through the blood to the genitourinary tract. Active genitourinary tuberculosis presents 5 to 25 years after the primary infection. So it is uncommon in young children. Eight to 15% of patients with pulmonary tuberculosis are supposed to be at risk of developing GUTB.[4] Patients may normally present with symptoms referred to the organ involved or may have long-standing, unexplained urological symptoms. The usual frequency of organ involvement is: kidney, bladder, fallopian tube, and scrotum.[5] The GUTB has varied presentation and some of the common ways are:

1. Recurrent or resistant urinary tract infection, sterile pyuria with or without hematuria.[6]
2. Irritative voiding symptoms, i.e., frequency, urgency, and dysuria.[6]
3. An incidental diagnosis in a known case of tuberculosis.
4. Renal (hydronephrosis/pyonephrosis) or epididymal mass.[7]
5. Infertility and pelvic inflammatory disease.[8]
6. Renal failure (Chronic kidney disease due to parenchymal infection and obstructive uropathy.[9]
7. The various other ways of presentation described are: flank pain with acute pyelonephritis, non-healing wounds, sinuses, or fistulae (nephrocutaneous fistula or vesicovaginal fistula), and hemospermia.[5–9]

The most common symptoms with which the patients have presented are in the form of irritative voiding, which are found in more than 50% of the patients. The other symptoms in GUTB can be fever, weight loss, anorexia, backache, and abdominal pain.[10]

## KIDNEY AND URETER

**Figure 1:**
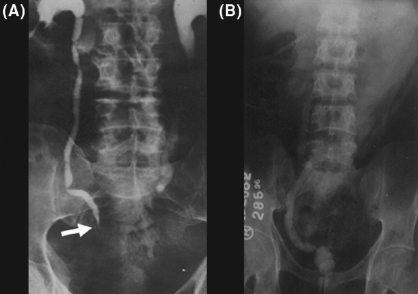
(A) Intravenous urography showing lower ureteric stricture (arrow head). (B) Cystogram showing thimble bladder with massive vesicoureric reflux

The disease can be advanced and of long-standing nature even with very few symptoms. Many a times, the patient is asymptomatic, but may have chronic sterile pyuria. Gross hematuria is seen in only 10%, but microscopic hematuria is present in up to 50% of the cases.[11] Acute renal pain is rare. It usually occurs secondary to luminal obstruction by blood clots, sloughed renal papilla, or flakes of calcification. Chronic dull ache may be due to infundibular, pelviureteric, or ureteric stricture [Figure 1A]. Some patients present with chronic renal failure which can be either due to renal parenchymal destruction secondary to infection or due to obstructive uropathy.[9]

## URINARY BLADDER

Involvement of the bladder is usually secondary to renal infection and is found in nearly one-third of the patients.[12] In the early stage, i.e., acute phase, bladder changes are usually non-specific which gives rise to irritative voiding symptoms. Chronic inflammation causes reduced compliance and capacity manifesting as frequency of micturition. Urgency develops if the bladder is extensively involved. Those who develop “thimble bladder” (due to mural fibrosis and contracture) [Figure 1B] may present with urinary incontinence.[13] Chronic inflammation and extensive fibrosis at vesicoureteric junction result in “Golf-hole ureter”.[14]

## PROSTATE, PENIS, AND URETHRA

These organs are uncommonly involved. Tuberculous prostatitis and urethritis can cause “beefy redness” and superficial ulcerations on endoscopic examination. Dilatation of the prostatic urethra, and “golf hole” dilatation of the prostatic ducts have also been reported.[15] Tuberculosis of the prostate may cause nodularity on digital rectal examination (DRE) mimicking malignancy. Sometimes, the diagnosis is made after histopathological examination (HPE) of transurethral prostatectomy (TURP) chips. Very rarely, fulminating prostatic involvement can cause abscess formation and subsequent perineal fistulization.

Primary involvement of the penis may have varied presentation. It may manifest as an ulcer clinically indistinguishable from sexually transmitted diseases (STDs) or malignancy. Rarely, it can cause cavernositis and cold abscess formation presenting as penile deformity or impotence.[16] Urethral involvement leads to stricture formation causing deterioration of the urinary stream and retention. Hematospermia, although rare, may be one of the presentations of genital tuberculosis. The incidence of hematospermia has been reported in up to 11% of the cases.[17]

### Epididymis and testes

Here, infection usually starts from the globus minor, as it has a richer vascularity. Usually presents as painful scrotal mass, which initially cannot be distinguished clinically from epididymo-orchitis. Sometimes, orchitis and the resulting testicular swelling can be difficult to differentiate from other mass lesions of the testes. These cases can present as infertility due to epididymal and/or vasal obstruction.[8] Nodular beading of the vas is a characteristic physical finding. Neglected cases may present as scrotal sinus.[18]

### Pelvic disease in females

The association of tuberculosis and pelvic disease most frequently presents as infertility, chronic pelvic pain, alterations in the menstrual pattern, or amenorrhea.[19]

### Adrenal glands

Adrenocortical insufficiency may result from involvement of the adrenal glands. Bilateral involvement of the adrenals causes their complete destruction (necrosis), resulting in adrenal insufficiency. In classic Addison's disease, tuberculosis is inactive and the adrenocortical tissue is replaced with granulomas, which is often partially calcified. Reactivation tuberculosis may result in signs and symptoms of tuberculosis along with the development of chronic adrenal insufficiency.[20]

### Diagnosis tests

Definitive diagnosis of tuberculosis involves demonstration of *M. tuberculosis* by microbiological, cytopathological, or histopathological methods (demonstration of granulomatous lesion). The usual tests used to diagnose GUTB are the demonstration of mycobacterium in urine or body fluid, and radiographic examination.[21]

### Urine examination

Sterile pyuria is the classic finding. Conventionally, demonstration of mycobacterium in urine has been used as the primary test for the diagnosis of GUTB. Preferably, five consecutive early-morning specimens of urine should be examined.[21] The yield of urine examination by smear and culture for detecting the tubercle bacillus is low, probably because of the intermittent shedding of the bacilli and is also observer-dependent. Direct smears are often negative (positive only in 30%) and urine cultures require 6 to 8 weeks in special culture media (Lowenstein-Jensen).[22] Sensitivity of urine culture in conventional cultural media is between 80 and 97%.[23]

## RAPID IDENTIFICATION OF MYCOBACTERIUM

### Radiometric systems

Radiometric liquid culture systems (i.e., BACTEC®) [Becton Dickinson, USA]) give rapid results and are highly sensitive in the identification of mycobacterium. But these methods have some inherent difficulties in working with radioactive materials, and the necessary apparatus used are really expensive. Recently, alternative growth detection methods for liquid culture employing oxygen quenching and redox reagents have been described and commercialized, which show performance comparable to BACTEC.[23] The time needed for culture and drug sensitivity testing is about 2 to 3 weeks.

### Polymerase chain reaction

Polymerase chain reaction (PCR) lets the sequence of DNA fragment from just a few mycobacteria to be amplified *in vitro* such that the amount of amplified DNA can be visualized and identified. The technique of PCR is rapid, with results available within few hours of DNA extraction from the sample. It is highly specific (up to 88%) and its sensitivity in detecting urine acid-fast bacilli (AFB) has been reported in up to 94% of the cases.[22,23]

### Imaging

In spite of the fact that the definitive diagnosis of genitourinary tuberculosis is established by positive results on urine culture or histologic examination, the imaging modalities make an important part of the investigation module. In the initial work-up, plain X-ray abdomen and chest are the two basic imaging tests. Plain X-ray abdomen may show renal calcification. Renal calcification may develop in 7 to 14% of patients.[6] Calcification rarely occurs in ureter (intraluminal), bladder wall, or seminal vesicles. Plain radiographs of chest and spine should be done to detect any pulmonary (active or old healed granuloma) or spinal involvement. Ultrasonography is a poor modality to show morphological changes. It is useful as an office procedure to monitor the degree of hydronephrosis/renal lesions during medical treatment. It also gives information regarding the bladder volume.

### Intravenous urography

**Figure 2:**
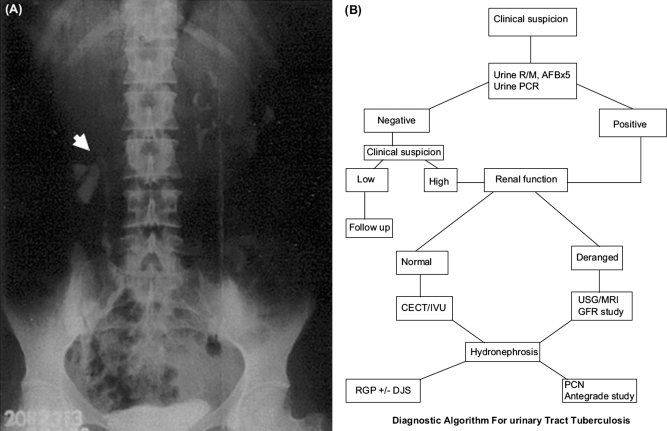
(A) Intravenous urography showing caliceal cut-off (arrow head). (B) Simple diagnostic algorithm in suspected cases of GUTB.[6,10,22,24,26–27]

Among the various imaging modalities, intravenous urography (IVU) has been considered to be one of the most useful tests. IVU provides both anatomical as well as functional details of the kidneys and ureters. The earliest radiographic changes of GUTB may demonstrate changes in the minor renal calyces with loss of sharpness and blunting. Progression of the disease will cause “moth eaten” appearance of the calyces and lost calyx due to infundibular stenosis [Figure 2A].[24]

Ureters when involved, these are initially dilated or become irregular in appearance. This is followed by stricture formation with predilection for the infundibulum, ureteropelvic junction, and distal ureter, which are the sites for narrowing. At these states, the ureter looks like beaded or pipestem or even corkscrew configuration. Ten to 56% of the patients develop ureteric strictures.[25] Involvement of ureteric orifices in the late stages, as these become fibrotic and fixed, leads to the development of vesicoureteric reflux (VUR).

### Computed tomography (CT scan)

Currently, at many centers, CT is replacing IVU as an imaging modality of choice in GUTB. It is equally good at identifying calyceal and infundibular abnormalities, renal parenchymal destruction, and hydronephrosis or hydroureter. In addition, it identifies adjacent adrenal, retroperitoneal, prostatic, and seminal vesicle abnormalities.[25]

### Cystoscopy and biopsy

Cystoscopy is rarely indicated for diagnostic purpose. Biopsy is needed if there is suspicion of malignancy. It should be done only after 4-6 weeks of medical therapy to prevent dissemination of the disease (i.e., tubercular meningitis). The cystoscopic findings are reduced bladder capacity and patulous ureteral orifice. The positive bladder biopsy diagnostic of GUTB can be found in up to 46% of the patients.[22]

### Retrograde and antegrade pyelography

There are two indications for it. One is for ureteral catheterization to obtain urine sample for culture for localization of the disease. The other indication being to delineate the stricture of the lower ureter. Percutaneous antegrade access is required if retrograde access is unachievable or insufficient for drainage of the kidney. It also provides a route for obtaining urine samples from the renal pelvis or tuberculous cavities for culture and to assess therapeutic drug concentration at the target sites.[26]

### Magnetic resonance imaging (MRI)

Useful in patients with compromised renal function, pregnancy, or allergy to contrast media. It gives good morphological details for the kidneys as well as excellent delineation of the ureters.[27]

### Radioisotope studies

Radioisotope studies are useful to determine split renal function and drainage in advanced stage of the disease.

We have earlier published our experience on GUTB, where we described a simple criteria to reach the definitive diagnosis of GUTB. Here, we made our diagnosis of GUTB on the basis of the presence of one major and/or two minor criteria. The major criteria were granulomatous lesion on histopathology, AFB positivity in urine or histopathology, and a positive PCR. Minor criteria included changes suggestive of tuberculosis on IVU/CT or MRI, hemaruria, raised ESR, and/or pulmonary changes of old healed granulomas. A simple diagnostic algorithm is presented through Figure 2B.
